# Supplementary material for: First laboratory confirmation and sequencing of Zaire ebolavirus in Uganda following two independent introductions of cases from the 10th Ebola Outbreak in the Democratic Republic of the Congo, June 2019
Source: PLoS Negl Trop Dis. 2022 Feb 22;16(2):e0010205. doi: 10.1371/journal.pntd.0010205 (PMC8896669; doi:10.1371/journal.pntd.0010205)
Supplement: Attachment — Submitted filename: Response_PLos Reviewer Comments_2.docx [file pntd.0010205.s001.docx]

Dear Editor, PLOS Neglected Tropical Diseases

Thank you for your consideration of our manuscript entitled;
 **"First Laboratory Confirmation and Sequencing of Zaire ebolavirus in Uganda after introduction of cases from the 10th Ebola Outbreak in the Democratic Republic of the Congo, June 2019**." We appreciate the feedback from all the reviewers that we have used to improve their current draft we are submitting. We have attended to all comments from the reviewers and the point-by-point response is as attached below. Also attached are fires of both clean and tracked changes copies of the manuscript. We shall be grateful for your positive response.

Yours sincerely

Dr. Luke Nyakarahuka, corresponding author.

Reviewer #2: The objectives are clearly articulated and the study design is appropriate. The population clearly described and appropriate.
Sample size or statistical analyses do=not apply. Ethical or regulatory requirements were met.

**Response: We thank the reviewer for the observation. We agree that sample size or statistical analysis do not apply in this case as it is a public health outbreak response as opposed to being a planned a research study. We followed all of the Uganda National task force on outbreak requirements for public health response as stipulated by the Uganda national task force and other international health regulations.**

Reviewer #2: The results are well presented.

**Response**: **We thank the reviewer for this response and feedback.**

Reviewer #2: The conclusions are well supported by the data. Limitations are clearly described. The usefulness and public health relevance are adequately discussed.

**Response:** **We thank the reviewer for this response and feedback.**

Reviewer #1: The methods section should precise which Elisa kits were used and the Ebola antigen that were used or targetted in the Elisa assays. The validation criteria (cut-off setting) should be explicite as well if the kit is an in-house one. A reference describing the optimisation of the Elisa assays can also be added.

**Response: For this study we used in-house ELISA assays developed and in use at CDC for over 20 years. The details of the method can be found in MacNeil et al 2011 (Serologic cross-reactivity of human IgM and IgG antibodies to five species of Ebola virus PLOS Neglected Tropical Diseases 5(6): e1175). Additional references to the development of the method can be found in Ksiazek et al. 1999 The Journal of Infectious Diseases 179(Suppl 1): S192-198), Ksiazek et al 1999 The Journal of Infectious Diseases 179(Suppl 1): 177-187 and Onyango et al 2007 The Journal of Infectious Diseases 196(Suppl 2): S193-S198. Briefly, both the IgM and IgG ELISA assays utilize inactivated, sonicated Ebola infected Vero E6 cells (positive-antigen) as the source of Ebola antigen; inactivated, sonicated non-infected Vero E6 cells (negative-antigen) are used to control for the Vero E6 background. Each patient serum sample is tested at four 4-fold dilutions, covering the range from 1:100, 1:400, 1:1600 and 1:6400. At each titration point the negative-antigen reading is subtracted from the positive-antigen reading to provide an adjusted optical density (OD410nm) reading for that titration point. Adding together all of the adjusted OD410nm readings for each titration provides a Sum OD410nm value. For a sample to be considered IgM positive, it must have a minimum titre of 1:400 (an IgM titre is considered positive if the adjusted OD410nm is >0.1) and a minimum Sum OD410nm value of >0.45. For a sample to be considered IgG positive, it must have a minimum titre of 1:400 (an IgG titre is considered positive if the adjusted OD410nm is >0.2) and a Sum OD410nm value of >0.95. This response has been added in manuscript and this methods section changed accordingly.**

Reviewer #1: In addition, the method section should mention the place of other investigations (for example the one which led detection of viruses such as CCHF, RVF...) and present the algorithm.

**Response: All samples submitted from all parts of the country including those from high-risk areas are subjected to PCR as a gold standard for testing VHFs. All samples submitted are tested for diagnosis of six viral species that cause VHFs which include Sudan Virus, Ebola virus, Bundibugyo virus, Marburgviruses, Crimean-Congo Hemorrhagic Fever virus and Rift Valley Fever Virus. This is how other outbreaks of VHFs were detected alongside surveillance for EVD. This has been added in the manuscript**

Was the Elisa done only for the PCR positive samples?

**Response: ELISA was performed on PCR positive samples and convalescent patients or those who were in very close contact with confirmed cases.**

Reviewer #2: The abstract should be revised for length and clarity.
 **Response: Done**

Reviewer #1: This nice paper is brilliantly written and illustrates how complementary are epidemiological investigations and lab testing or sequencing. It also points out the impact of preparedness program and the role of countries collaboration when faciing a commun threat.

**Response**: **We thank the reviewer for this response and feedback.**

Reviewer #2: Nyakarahuka and coworkers have done a terrific job of detailing laboratory confirmation and sequencing of Ebola virus in Uganda after it was introduced from the Democratic Republic of the Congo. The detail of the epidemiology is striking and important work.

This reviewer has only a few suggestions:

1.      Please revise the abstract – it is too long for PLOS NTD and misses the guidance to “summarize the most important results with important numerical results given.” Some of these results are summarized nicely in the last paragraph of the paper. It seems important that “the case detected later in August 2019 (201901815) was from a different lineage from the three related cases in June 2019, demonstrating that the two Uganda EVD outbreaks were independent introductions and not the result of ongoing EBOV transmission in Uganda.”

Response: **The abstract has been revised to meet PLOS NTD Requirements with a word count of 200.**

2.      With regard to the two independent introductions, it might be more accurate to revise the title of the paper a bit.

**Response. This has been put into consideration and the title edited accordingly.**

3.      Apologies, but please revise to confirm to the current nomenclature rules with respect to capitalization and use of italics. Also, species of viruses don’t cause disease, members of species do. The title should probably just say Ebola virus, not the species name.
**Response. We thank the reviewer for this comment. Because this species of a Ebolavirus was being detected in Uganda for the first time we thought to emphasize this in the title. Since the name Ebola virus has been used synonymously for other species of genera by the general public, using the real species name in the title would help us communicate the significance of this finding.**
--------------------
